# Adult zebrafish as a model organism for behavioural genetics

**DOI:** 10.1186/1471-2202-11-90

**Published:** 2010-08-02

**Authors:** William Norton, Laure Bally-Cuif

**Affiliations:** 1 Zebrafish Neurogenetics group, Laboratory of Neurobiology & Development (NED), CNRS, UPR 3294, Institute of Neurobiology Albert Fessard, Avenue de la Terrasse, 91198 cedex, Gif-sur-Yvette, France

## Abstract

Recent research has demonstrated the suitability of adult zebrafish to model some aspects of complex behaviour. Studies of reward behaviour, learning and memory, aggression, anxiety and sleep strongly suggest that conserved regulatory processes underlie behaviour in zebrafish and mammals. The isolation and molecular analysis of zebrafish behavioural mutants is now starting, allowing the identification of novel behavioural control genes. As a result of this, studies of adult zebrafish are now helping to uncover the genetic pathways and neural circuits that control vertebrate behaviour.

## Review

Henry David Thoreau wrote "Many men go fishing all of their lives, without knowing it is not fish they are after". Thus, one of the intrinsic difficulties of studying behaviour is to understand the underlying motivation of complex behaviours. Most behavioural traits are multigenic and display environmental interactions, further compounding the difficulty of analysing them. However, recent studies using rats, mice, zebrafish, nematodes and fruit flies have begun to identify the genetic toolbox that controls behaviour.

The general suitability of zebrafish as a model organism, as well as its use in the genetic and neuroanatomical analysis of larval behaviour has been comprehensively described elsewhere [1,2]. Although more difficult to manipulate than larvae, adult zebrafish display a full repertoire of mature behaviours making their characterisation particularly enticing. Zebrafish (*Danio rerio*) are a typical cyprinid (carp family) schooling fish. In contrast to other laboratory behavioural models, zebrafish are naturally social animals that show preference for the presence of conspecifics [3,4]. Zebrafish are therefore an excellent model to probe the genetics of social behaviour. In addition, zebrafish are diurnal allowing behaviour to be measured during their natural day time. Finally, it is crucial to investigate whether complex behaviours such as reward, learning and social behaviour are conserved throughout the animal kingdom. Thus, comparative studies of many model organisms, including zebrafish, are necessary to determine general principles of behavioural control. Several groups have already developed protocols to measure aggression, alarm reaction, antipredatory behaviour, anxiety, locomotion, learning and memory, sleep, reward and social behaviour (see Table 1 and references therein). In this review we consider the brain areas and neurotransmitter systems that have been linked to the control of behavioural in adult zebrafish. We also describe the protocols and tools that have been developed for zebrafish behavioural studies.

**Table 1:** Protocols to measure behaviour in adult zebrafish.

Stage	Behaviour	Paradigm	Reference
Adult	Aggression	Live observation of two fish	[29,37,39,40,45]
Adult	Aggression	Mirror image test	[6]
Adult	Aggression	Pigment response	[6]
Adult	Aggression	Startle reaction	[85]
Adult	Alarm reaction	Response to alarm substance	[26,52,86]
Adult	Antipredation	Predator stimulation	[87]
Adult	Anxiety	Exit latency test	[51]
Adult	Anxiety	Group preference	[6]
Adult	Anxiety	Light/Dark preference	[48,88]
Adult	Anxiety	Locomotory activity	[6]
Adult	Anxiety	Place preference / Thigmotaxis	[13,34,49,51]
Adult	Anxiety	Tank diving test	[50,53,55]
Adult	Anxiety	Time in enriched T-maze chamber	[89]
Adult	Audition	Response to startling noise	[64]
Adult	Courtship	Observation of courtship postures	[82]
Adult	Lateralisation	Interaction with object	[41,77]
Adult	Locomotion	Mean velocity	[90]
Adult	Locomotion	Number of lines crossed	[36,81,87]
Adult	Locomotion	Total distance moved / Videotracking	[80,81,90]
Adult	Locomotion	Turning angle	[90]
Adult	Mate choice	Video-stimulus technique	[91]
Adult	Learning / memory	Active avoidance conditioning	[27,28,92,93]
Adult	Learning / memory	Delayed spatial alternation	[35]
Adult	Learning / memory	Learned alarm reactions	[86]
Adult	Learning / memory	Spatial alternation, learning and memory	[31,32,54,94]
Adult	Learning / memory	T-maze	[8,13,33,89]
Adult	Learning / memory	Visual discrimination learning	[29]
Adult	Olfaction	Response to amino acids	[75]
Adult	Reward	Conditioned place preference	[6-9,13]
Adult	Reward	Presence of Conspecific	[11]
Adult	Sleep	Locomotor inhibition	[61]
Adult	Sleep	Monitoring sleep postures	[57,58,63]
Adult	Sleep	Pigment response	[61]
Adult	Social preference	Area occupied	[85]
Adult	Social preference	Group preference	[6]
Adult	Social preference	Nearest neighbour distance	[85]
Adult	Social preference	Shoaling	[3,78,87]
Adult	Vision	Optokinetic response	[76]

### Contributions of zebrafish to behavioural genetics: Reward and Learning

#### Reward behaviour

Perhaps the most prominent area in which the adult zebrafish has contributed to behavioural genetics is reward. Reward behaviour provides animals with an instinctive drive to search for resources and to reproduce. However, the brain's reward pathway can also be hijacked by drugs of abuse such as cocaine, amphetamine or opioids. Reward behaviour may thus constitute the first step towards addiction. Reward can been measured in zebrafish by using the conditioned place preference (CPP) test, which pairs a primary cue (e.g. a drug) with a secondary stimulus such as a coloured aquarium compartment. Drug dependency can also be evaluated by measuring the persistence of CPP following a period of abstinence.

In line with studies of other animals (e.g. [5]), stimuli that have been shown to be rewarding for adult fish include ethanol [6,7], cocaine [8], amphetamine [9], opiates [10], nicotine [7], food [10] and the presence of conspecifics [11]. The major neurotransmitter associated with reward behaviour is Dopamine (DA). Increases of DAergic signalling from the ventral tegmental area to the nucleus accumbens (nAC) motivates mammals to repeat stimulus application. In zebrafish, this key DAergic pathway is most likely comprised of projections from the diencephalic posterior tuberculum to the ventral telencephalon (subpallium, (Vv and Vd), see [12]). Several other neurotransmitters have also been implicated in reward behaviour. Heterozygous mutant zebrafish lacking one copy of the *acetylcholinesterase *(*ache*) gene have enhanced acetylcholine levels in the brain due to decreased breakdown of the neurotransmitter. The increase of acetylcholine in the brain of *ache *mutants causes a decrease in amphetamine-induced CPP [13]. Mammalian reward pathways also include raphe 5-HTergic neurons [14] as well as a number of inhibitory influences including projections from the habenula. The zebrafish ventral habenula appears to be homologous to the mammalian lateral habenula in both gene expression and innervation of the raphe [15]. The recent identification of selective molecular markers for both structures [16,17] will make genetic manipulation of the reward pathway possible. Such a targeted approach will allow functional interrogation of the reward circuitry in zebrafish and may highlight both similarities and differences in the mechanisms controlling monoaminergic behaviours in vertebrates.

There have been several screens for zebrafish mutant families with altered reward behaviour. Darland and Dowling isolated three families of mutants which were not responsive to cocaine application, although the affected genes were not reported [8]. Other groups have used microarrays to identify addiction related genes. Brennan and colleagues demonstrated a robust change in place preference (PP) following nicotine or ethanol treatment [7]. This PP was also maintained following a period of abstinence or when paired with an adverse stimulus (3 seconds removal from the tank water) suggesting that drug dependency had occurred. Microarray analysis comparing the brains of both treated and untreated fish identified 1362 genes that were significantly changed following drug application, including 153 that were responsive to both nicotine and ethanol. Many of these genes are also involved in reward behaviour in other species, by either altering dopaminergic or glutamatergic signalling or modulating synaptic plasticity [7]. In addition, this study also revealed a number of novel genes that were changed upon drug administration, including those coding for Calcineurin B and the Hypocretin receptor. Bally-Cuif and colleagues conducted a screen for mutants that were insensitive to amphetamine application [13]. One of these mutants, *no addiction *(*nad*), was used to identify genes that were transcriptionally modified by amphetamine in wild-type fish and were differentially over- or under-regulated in *nad*. Importantly, gene expression was unmodified in *nad *mutants in the absence of the drug [9]. This strategy permitted the unbiased recovery of 139 genes linked to amphetamine triggered CPP. A large proportion these genes were developmentally active transcription factors. These include Dlx1a, Emx1a, Lhx8, Sox9 and Tbr1, proteins that are implicated in the control of neurogenesis in the vertebrate embryo and show persistent expression in the adult-neurogenic regions of the mammalian and fish brain [9]. A recent study of rats has also demonstrated altered cocaine (but not sucrose) mediated reward behaviour following a reduction of hippocampal neurogenesis [18]. This constitutes an exciting new development in the field of reward behaviour; neurogenesis-induced plasticity may account for some of the learning aspects of reward and the long-lasting changes in the brain associated with addiction.

A comparable approach has led to the identification of *too few *(*tof*) a mutant that fails to change place preference following morphine treatment but not food application [10]. *tof *encodes a forebrain specific zinc finger protein, Fezl [19], which establishes *neurog1*-expressing DA progenitor domains in the basal forebrain [20]. Loss of *fezl *leads to a reduction of dopaminergic and serotonergic neurons in specific nuclei of the forebrain (diencephalon and hypothalamus; [21]), defects that are maintained into adulthood. Dissociation between the preference for a natural reward (food) and a drug (morphine) has previously been observed in dopamine D2 receptor knock-out mice [22] but is not understood at the molecular or neurological level. Since both morphine and food rewards are dependent on opioid receptor activity in zebrafish [10], the separable reward behaviour seen in *tof *suggests that distinct neural systems act downstream of opioid signalling to mediate the response to morphine and food. Alternatively, the rewarding aspects of food and drug treatment may be mediated by different subsets of dopaminergic nuclei in the forebrain. Thus, together with the study of hippocampal irradiated rats [18], *tof *presents an excellent opportunity to dissect the neural basis of discrimination between rewarding substances in the brain.

#### Learning and memory

Studies of mammals have shown that learning and memory can be controlled by several brain circuits, each of which is neuroanatomically distinct. These include spatial learning (hippocampus), implicit learning (such as simple motor reflexes; cerebellum) and avoidance learning (amygdala). Although the neural basis of learning is not well understood in zebrafish, studies of the closely related goldfish (*Cassius auratus*) hint at brain areas which could be involved. Focal ablations of the goldfish brain have identified the lateral pallium (Dl, equivalent to the hippocampus), medial pallium (Dm, equivalent to the amygdala) and cerebellum [23-25] as playing key roles in learning.

Several paradigms have already been developed to measure learning and memory in zebrafish. Associative learning can be measured by pairing two previously unrelated stimuli such as colour, reward or aversion. For example, Suboski and colleagues paired a neutral stimulus (morpholine) with the aversive effects of alarm substance [26]. Avoidance learning can be assessed by using a shuttle box; fish are quickly able to associate a conditioned stimulus (e.g light [27] or colour [28]) with an unconditioned stimulus (such as a mild electric shock). Spatial learning can be measured using either a T-maze [8,11,29,30] or a shuttle box [31,32]. Fish must learn to collect a reward by either navigating a maze correctly or alternating the side of the tank visited. These behavioural tests are high-throughput, making them suitable for screens for novel genes controlling learning and memory.

Pharmacological studies have validated adult zebrafish as a model for learning and memory, making it a very promising area for future research. Several evolutionarily conserved neurotransmitter systems have been implicated in learning and memory. *ache *mutants, with increased acetylcholine levels in the brain, learn to find a food reward faster in a T-maze [33] thereby linking cholinergic signalling to learning. Fish exposed to moderate levels of nicotine perform better in a delayed spatial alteration task, a type of avoidance learning test. Nicotine acts via nicotinic acetylcholine receptors (nACHRs), thus further underscoring the importance of cholinergic signalling [34,35]. Treatment of fish with a Histidine decarboxylase inhibitor, alphafluoromethylhistidine (α-FMH), reduces both levels of histamine and the number of histaminergic fibres in the brain. α-FMH treated animals display defects in long-term memory formation but not initial learning [30]. Finally, NMDA antagonists have also been shown to impair memory formation in zebrafish [27,28]. NMDA receptors are found abundantly in the telencephalon, which contains the teleostean (bony fish) equivalent of the hippocampus and amygdala [36]. The neurotransmitters discussed above have also been connected to learning in other species, suggesting that work in zebrafish may give insight into conserved learning mechanisms. Therefore, adult zebrafish constitute a particularly promising model for research into learning and memory.

### Emerging fields for genetic analysis: Aggression and Anxiety

#### Aggression

Aggression is a complex suite of behaviours serving a number of adaptive purposes. Fish use aggression to protect offspring, monopolise resources such as food, territory and mates and establish dominance hierarchies. Aggression can be measured in the laboratory by recording the interaction of two free-swimming fish or by using mirror induced stimulation (MIS)[6,37]. Fish are unable to recognise their own image and so attack as if an intruder is present [38]. Furthermore, MIS provides immediate feedback to the fish's activity and avoids damaging the subjects [37]. Zebrafish display characteristic agonistic postures including erection of the dorsal, caudal, pectoral and anal fins coupled to biting, thrashing of the tail and short bouts of fast swimming directed against the mirror [6]. A positive correlation between aggression and boldness has also been reported [39]. Aggression is a very plastic behaviour. Both habitat complexity and rearing conditions can influence the number of interactions [37,40]. Furthermore, different wild-type strains show varying aggression levels suggesting a genetic component to its control. Finally, aggressive behaviour also shows lateralisation, with adult fish predominantly using the right eye to view predators [41].

Studies in other species have identified 5-HT as the major neurotransmitter controlling aggression. Animals with high levels of 5-HT tend to be timid, whereas those with lower levels are more impulsive and aggressive (e.g. [42]). Other neurotransmitters, including GABA, glutamate and nitric oxide as well as the hormones vasopressin and testosterone have also been implicated in agonistic behaviour [43]. However, the role of these neurotransmitters has not been explicitly tested in zebrafish. In zebrafish, agonistic behaviour can be modified by exposure to pharmacological compounds including ethanol [6] and 17alpha-ethinylestradiol (a synthetic oestrogen; [44]). The brain areas mediating aggression in fish are not well characterised. Arginine vasotocin-expressing cells of the magnocellular preoptic area change size depending on the dominance status of fish. This suggests involvement of the preoptic area in control of social hierarchy and the agonistic behaviour used to establish it [45]. Studies of other fish species have identified additional brain territories that influence aggression. For example, the neural activity maker *cfos *is expressed in the diencephalon, thalamus and hypothalamus and a few nuclei in the pons and medulla oblongata of the mudskipper (*Periophthalmus cantonensis*) following an aggressive episode [46]. Finally, electrical stimulation of the bluegill (*Lepomis macrochirus*) implicates the inferior hypothalamus in aggression control [47].

The MIS protocol is simple to establish and perform. Coupled to computer-aided automation, it can be adapted for high-throughput screening studies, thus providing a golden opportunity to uncover novel genes implicated in aggression control.

#### Anxiety

Anxiety is a state of constant fear or restlessness caused by anticipation of a real or imagined future event. Multiple anxiety tests have been established in fish, although it is not always clear whether fear or anxiety is being measured, or indeed whether the different states even exist [48]. Protocols to measure anxiety tend to assess one of two variables. The first set of protocols record the reaction of adult fish to novel environments, such as the amount of time spent at the edge of a tank [30,49], at the bottom of a novel tank [34,50] or on the dark side of a light/dark tank [28,51]. The second approach analyses locomotory patterns: freezing, long-lasting increases in basal locomotory activity [6,48,49] and tightening of a fish's shoal [52] have all been reported to be reliable measures of anxiety. The expression and level of anxiety are wild-type strain dependent [49,50]. For example, AB wild-types manifest anxiety as a hyperactive swimming response [49].

Similar to other behaviours, anxiety protocols have been validated using pharmacological compounds developed for human patients. Application of caffeine [50,53], pentylenetetrazole [53], alarm substance [50,52], the benzodiazepine partial inverse agonist FG-7142 [49] and withdrawal of cocaine [49] have all been shown to be anxiogenic. Conversely, many anxiolytic substances have been characterised including nicotine [54], diazepam [49,55], the Htr1a (5-HT receptor) partial agonist buspirone [55], fluoxetine hydrochloride and ethanol [50]. Finally, a link between anxiety levels and the major zebrafish stress hormone cortisol has also been demonstrated [50]. The ease of applying drugs and robust behavioural assays (see [49,50]) make zebrafish an ideal model to study anxiety and related behaviours.

### Sleep

Although sleep is a widespread phenomenon, its behavioural and physiological function is not well understood. Sleep is characterised by periods of behavioural quietness, species-specific body postures, an increased arousal threshold and a quick return to wakefulness [56]. Furthermore, sleep-deprived animals also show homeostatic rebound, increasing the amount of time needed to sleep following deprivation. The timing of sleep also shows circadian rhythmicity. Several studies have identified sleep-like behaviour in zebrafish. During the night, adult fish have periods of two to four minutes of inactivity in which the fish floats horizontally and makes small pectoral fin movements. There is also a simultaneous reduction of mouth and operculum movements suggesting lower respiratory levels [57]. Sleep rebound has been demonstrated in zebrafish indicating homeostatic regulation; disrupting the normal night time routine (using light, vibration, electric shock or forced movement) deprives fish of rest and causes a subsequent increase in sleep duration [57,58]. Finally, zebrafish also show circadian rhythmicity, with higher activity levels in the day [57].

Studies in other species have identified several significant sleep-related neurotransmitters: Increases of dopamine levels in the brain reduces the amount of time asleep [59], whereas GABA signalling promotes sleep and GABA_A _receptor agonists are used to treat insomnia [60]. Although these pathways have not been directly examined in zebrafish, treatment with diazepam, pentobarbital [57], alpha2 adrenoceptor agonists [61], and histamine H1 antagonists [62] have all been shown to increase sleep, thus implicating GABA, acetylcholine and histamine in its control. Several studies have also demonstrated a conserved role for hypocretin/orexin (HCRT) in sleep-wake regulation. Zebrafish contain a single HCRT receptor gene (*hcrtr*), which is expressed in a small number of glutamatergic neurons of the adult hypothalamus [58,63]. Loss of *hcrtr *function causes sleep fragmentation but not cataplexy or decreased wake bout length, suggesting that HCRT may function to consolidate sleep in fish [58]. HCRT acts by stimulating the endogenous melatonin sleep-promoting system found in the pineal gland [63]. Taken together, studies of zebrafish have confirmed that the control of sleep appears to be evolutionarily conserved. Although zebrafish sleep research is still in its infancy, the high throughput nature of the set-ups used to measure sleep demonstrates that zebrafish are an ideal model in which to conduct screens for novel hypnotic mutants.

### Practical considerations: strain differences, screen design and duplicated genes

#### Strain differences in wild-type fish

The examples discussed in this review highlight the suitability of adult zebrafish for studies of complex vertebrate behaviours. However, there are several considerations that need to be taken into account before initiating behavioural work. For example, care must be taken to dissect the influence of neurotransmitter signalling pathways and the specificity of drugs used to modulate them. Finally, another important consideration when designing behavioural studies is the background strain of the fish used. Strain differences in adult behaviour have already been reported [13,49,50,64]. Thus, in order to avoid some of the known difficulties in reproducing behavioural work, all behavioural studies should be carried out on well defined laboratory strains. Although no inbred strains exist, the AB line, available from the ZIRC stock centre is an excellent choice for a reference strain. The line has been maintained in the laboratory for more than 70 generations and is freely available to the zebrafish community.

#### Screen design

**Figure 1:**
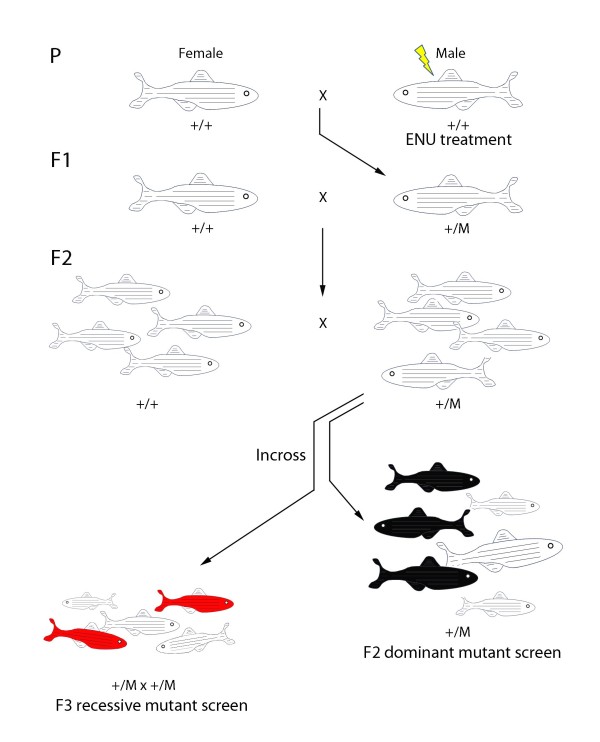
**Three-generation breeding scheme for chemically-induced mutant fish**. Male fish are mutagenised and then crossed to wild-type females to produce an F1 generation. An F2 generation is made by in-crossing F1 siblings. Dominant behavioural mutants can be identified in this F2 generation (black fish). For recessive mutant carriers, a second in-cross is performed and the progeny screened for behavioural alterations (red fish) - if the inheritance in Mendelian then one quarter of the progeny should show the behavioural defect.

**Figure 2:**
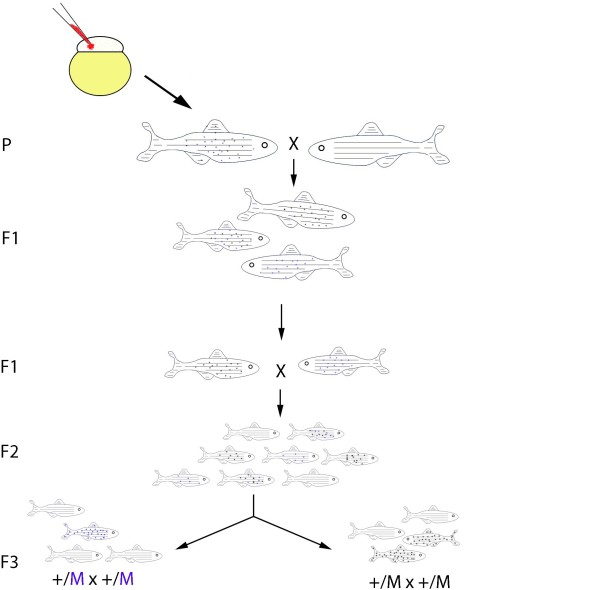
**Breeding scheme for the production of insertional mutants in zebrafish**. Single-cell to blastula-stage embryos are injected with a mutagen and grown to adulthood. The mature fish are then inbred twice to produce first an F1 and then an F2 generation. Mutants with behavioural phenotypes (black and blue spotted fish) can be identified by in-crossing the F3 fish. The number- and position of insertions can be monitored by western blot and PCR analysis.

Genes do not directly control behaviour. Rather, genes influence behavioural output by either modulating neural circuit formation (neural specification, differentiation and connectivity) or function (e.g. neurotransmitter release or reuptake). High throughput forward genetic screening has long been one of the goals of zebrafish research, and in this regard the nascent behavioural field is no different. However, behavioural phenotyping is subject to large variability between animals. This can make it difficult to phenotype mutants with certainty, and so complicates positional cloning of the mutations. Furthermore, careful consideration needs to be given to the choice of mutagen. The most commonly used mutagen N-ethyl-N-nitrosourea (ENU; Fig. 1) efficiently induces intragenic point mutations in the germline [65], but the subsequent cloning steps needed to recover the mutagenised gene are laborious. As an alternative to ENU treatment, insertional mutagenesis looks particularly promising (Fig 2). Although insertional mutations occur at a lower frequency, isolation of the genetic lesion is much simpler [66,67]. The mutagenic cassette may also be coupled to a fluorescent reporter line thus highlighting the expression profile of the mutated gene. This technique will allow faster and more reliable identification of animals carrying the same insertion and so will facilitate mapping. This has recently been powerfully demonstrated in juvenile fish by using a reporter-tagged insertional mutagenesis strategy to clone two nicotine-response mutants [68]. Finally, the usefulness of zebrafish is not limited to screening paradigms. The advent of TILLING [69] and zinc-finger nuclease technology [70] has opened the door to targeted modification of the zebrafish genome, thus allowing the behavioural function of known genes to be probed.

### Gene duplication and redundancy in zebrafish

In common with all ray-finned fishes (actinopterygii), zebrafish underwent a third whole genome duplication around 350 million years ago and often have two copies of genes found in other vertebrates [71]. The most likely fate of a duplicate gene is loss of function. However, in some cases both copies can be retained and subfunctionalisation (splitting of the ancestral function between the two new genes) or neofunctionalisation (acquisition of a new function through mutation) can occur [72]. Redundancy can make analysis of a gene's function more difficult by masking mutant phenotypes. However, redundancy can also be useful, exposing late functions of genes that cause embryonic defects in other animals. For example, zebrafish lacking activity of one copy of *fibroblast growth factor 1 *(*fgfr1a*) have a surprising lack of developmental phenotype compared to mice and medaka deficient in the gene [73,74]. Rather, adult *fgfr1a *mutant fish exhibit several behavioural alterations, including increases in aggression, boldness and exploration (W Norton, personal observation).

## Conclusion

Although anecdotally fish are thought to have poor memories and display few complex behaviours, numerous studies have disproved such beliefs. In this review we have demonstrated ways in which studies of adult zebrafish have contributed to our understanding of the genetic basis of behaviour. We have described set-ups to measure behaviour (e.g. Table 1) and some of the pharmacological treatments that have already been employed in zebrafish (Table 2). However, fish also manifest other behaviours, the discussion of which is unfortunately beyond the scope of this review. These behaviours include olfaction [75], vision [76], behavioural lateralisation [77], shoaling [3,78,79], locomotion [80,81] and reproductive behaviour [82]. Finally, studies of adult fish are also beginning to give clues about the initiation of locomotion, an assay that might be modified to probe the motivation to move. In the adult spinal cord, application of 5-HT modifies the cyclical pattern of locomotory activity by increasing mid-cycle inhibition and reducing the onset of the next cycle, so reducing the initiation of locomotion [83].

**Table 2:** Pharmacological treatments with known behavioural effects on adult zebrafish.

Behaviour	Modulating agent	Function / Activity	Effect	Reference
Aggression	Ethanol	GABA-A receptor modulator	Increases aggression	[85]
Aggression	17α-ethinylestradiol	Synthetic oestrogen	Reduces aggression	[44]
Antipredation	Ethanol	GABA-A receptor modulator	Impaired by high doses	[6]
Anxiety	Diazepam	Benzodiazepine	Reduces anxiety	[49,86]
Anxiety	FG-7142	Benzodiazepine inv. agonist	Increases anxiety	[49]
Anxiety	Pentylenetetrazole	GABA antagonist	Increases anxiety	[53]
Anxiety	Ethanol	GABA-A receptor modulator	Reduces anxiety	[50,53]
Anxiety	Buspirone	Htr1A partial agonist	Reduces anxiety	[55]
Anxiety	Alarm substance	Hypoxanthine-3N-oxide	Increases anxiety	[50,52]
Anxiety	Nicotine	NachR agonist	Reduces anxiety	[34]
Anxiety	Methyllycaconitine	Nicotinic antagonist	Anxiolytic	[55]
Anxiety	Dihydro-β-erythroidine	Nicotinic antagonist	Anxiolytic	[55]
Anxiety	Mecamylamine	Nicotinic antagonist	Anxiolytic	[34]
Anxiety	Morphine	Opiate	Reduces anxiety	[53]
Anxiety	Cocaine (withdrawal)	Psychostimulant	Increases anxiety	[49]
Anxiety	Fluoxetine	5-HT reuptake inhibitor	Reduces anxiety	[50,53]
Anxiety	Caffeine	Xanthine alkaloid	Increases anxiety	[50,53]
Group preference	Ethanol	GABA-A receptor modulator	Reduced at high conc.	[6]
Learning	α FMH	HDAC inhibitor	Impairs long term memory	[30]
Learning	Nicotine	NachR agonist	Improves learning	[34,35]
Learning	MK-801	NMDA antagonist	Impairs memory	[27,28]
Learning	L-NAME	NO synthase inhibitor	Impairs memory retention	[27]
Light/Dark pref	Ethanol	GABA-A receptor modulator	Decreased at high conc.	[6]
Locomotion	Ethanol	GABA-A receptor modulator	Reduced at high conc.	[6]
Reward	Acetylcholine	Cholinergic agonist	Non-rewarding	[13]
Reward	Ethanol	GABA-A receptor modulator	Rewarding	[7]
Reward	Nicotine	NachR agonist	Rewarding	[7]
Reward	Food	Nourishment	Rewarding	[10]
Reward	Morphine	Opiate	Rewarding	[10]
Reward	Morphine	Opiate	Rewarding	[10]
Reward	Cocaine	Psychostimulant	Rewarding	[8]
Reward	Amphetamine	Psychostimulant	Rewarding	[9]
Sleep	Dexmedetomidine	alpha2 adrenoceptor agonist	Sedative	[61]
Sleep	Pentobarbital	Barbiturate	Hypnotic	[57]
Sleep	Diazepam	Benzodiazepine	Hypnotic	[57]

Larval zebrafish are also useful for studying simple behaviours, and protocols have been established to measure locomotion and visuomotor behaviours such as prey capture [1]. Coupled to the transgenic lines available and the emergence of optogenetic technology (e.g. [84]), larvae may allow the dissection of behavioural circuits at the cellular level in intact living fish. Moreover, in an elegant recent study by Engert and colleagues, neural circuit activity has been analysed at the single-cell level by recording bioluminescence in free-swimming larvae [85].

In summary, zebrafish have many attributes that make them an ideal model organism for the study of behavioural genetics. Although to date there have been relatively few studies of adult zebrafish behaviour, the ease of carrying out pharmacological studies coupled to the ever increasing number of available genetic tools suggest that zebrafish are about to enter the limelight. Finally, their small size and cheap maintenance costs suggest that zebrafish are ideally suited for large-scale behavioural screens. We look forwards to the next steps in the establishment of this fascinating field.

## Authors' contributions

WN conceived the review and wrote the manuscript. LB-C suggested subject areas to include in the review and worked on the manuscript. All authors read and approved the final manuscript.
